# Solid Phase Extraction of Inorganic Mercury Using 5-Phenylazo-8-hydroxyquinoline and Determination by Cold Vapor Atomic Fluorescence Spectroscopy in Natural Water Samples

**DOI:** 10.1155/2013/134565

**Published:** 2013-12-26

**Authors:** Mirna Daye, Baghdad Ouddane, Jalal Halwani, Mariam Hamzeh

**Affiliations:** ^1^Université Lille 1, Equipe Chimie Analytique et Marine, UMR-CNRS 8217 Géosystèmes, Villeneuve d'Ascq Cedex, France; ^2^Water & Environmental Sciences Laboratory (L.S.E.E), Lebanese University, Lebanon

## Abstract

8-Hydroxyquinoline (8-HQ) was chosen as a powerful ligand for Hg solid phase extraction. Among several chelating resins based on 8-HQ, 5-phenylazo-8-hydroxyquinoline (5Ph8HQ) is used for mercury extraction in which the adsorption dynamics were fully studied. It has been shown that Hg(II) is totally absorbed by 5Ph8HQ within the first 30 minutes of contact time with *t*_1/2_ 5 minutes, following Langmuir adsorption model. At pH 4, the affinity of mercury is unchallenged by other metals except, for Cu(II), which have shown higher Kd value. With these latter characteristics, 5Ph8HQ was examined for the preconcentration of trace levels of Hg(II). The developed method showed quantitative recoveries of Hg(II) with LOD = 0.21 pg mL^−1^ and RSD = 3–6% using cold vapor atomic fluorescence spectroscopy (CV-AFS) with a preconcentration factor greater than 250.

## 1. Introduction

Mercury is a ubiquitous element and is one of the most toxic environmental pollutants. In aquatic environments, mercury exists mainly as three forms, which are elemental mercury (Hg°), inorganic mercury Hg(II), and organic mercury. While all forms of mercury are poisonous, methyl mercury is the most toxic species due to its capacity of bioaccumulation through the aquatic food chain. The most employed analytical methods for trace mercury determination are inductively coupled plasma mass spectrometry (ICP-MS), Cold Vapor-Atomic Absorption Spectroscopy (CV-AAS), and cold vapor atomic fluorescence spectroscopy (CV-AFS). In natural waters, mercury concentration is found at trace levels in which most analytical techniques fail in its direct determination. Subsequently, a separation/preconcentration step is often indispensable. Among many preconcentration methods, solid phase extraction (SPE) is the most used for trace metal extraction.

8-Hydroxyquinoline (8-HQ) has a complexing coordinating ability with over 60 metals with preference to transition metals over alkali and alkaline earth metals. It is even used for the extraction of rare earth metals using a binary mixture of sec-octylphenoxyacetic acid-8-HQ [1]. 8-HQ forms chelates with metals of varying stability. Log ⁡*K*_1_ values for Fe(III), Cu(II), Cd(II), Ca(II), and Mg(II) with 8-HQ are 13.7, 12.1, 7.3, 3.3, and 4.2, respectively [2]. Stability constant of Ca(II) with immobilized 8-HQ was also determined by [3] with slightly higher value of log⁡⁡*K*_1_ of 3.7. Moreover, Weaver and Harris [4] calculated the stability constant of aluminum with alkyl-immobilized 8-HQ and found it to be log⁡⁡*K*_1_ 0.8. High stability constants were found for Cd(II) with 8-HQ immobilized on controlled pore glass (CPG) ranging from 10^9^–10^11^ [5]. 8-HQ has been used extensively for the preconcentration of trace metals from natural samples and seawater. Silica has been used as a support in many applications for its numerous advantages as compared to polymers. 8-HQ has been immobilized on various solid matrices from XAD to activated carbon [6]. Adsorption capacities have been determined for some metals, that is, Cu(II) using 8-HQ functionalized CPG 58 *μ*mole/g, Porasil C porous silica 104 *μ*mole/g, and silica gel 216 *μ*mole/g. It is evident that silica gel confers the largest capacity with an average molar coverage of 0.39 *μ*mole/m^2^ attributed to increased surface area of 553.84 m^2^/g (decreased pore size) [7].

Numerous synthesis routes have been described since the first immobilization of 8-hydroxyquinoline (8-HQ) on silica gel. Diazo coupling of 8-HQ with p-aminobenzamide propyl silica was first reported in [23, 34], The first techniques were further ameliorated by diazo coupling of 8-HQ on p-amino phenyl silica [24] or glass [25]. Moreover, the formation of Schiff's base on position 5 of 8-HQ moiety enables its fixation on p-aminopropyl silica bestowing extraordinary coordination [26]. Finally, following the Mannich reaction, which offers two possible procedures of 8-HQ immobilization on silica phase; one involving two steps reactions [27, 28] and the other one-step reaction [29, 30]. Among these synthesis routes, immobilization involving the diazo coupling of 8-HQ on p-aminobenzamide propyl silica was shown by many studies to have an excellent retention of many trace metals. The most powerful chelating 8-HQ-grafted silica is that with an exceptional coordination potential given by its anchoring position on carbon 5 of 8-HQ moiety. Therefore, phenylazo-8-HQ was chosen in this study among other 8-HQ-5-grafted silica due its higher yield product.

Since then much research has been conducted to fully understand the complexing ability of phenylazo-8-HQ (5Ph8HQ). Spectroscopic studies of metal complexes with 5Ph8HQ bound to silica showed 1 : 1 metal ligand stoichiometry in Cu^2+^ solution complex [31]. Other studies have determined the protonation constants of 5Ph8HQ-grafted silica to be *pKa*_1_ = 2.7 and *pKa*_2_ = 8.6 [31]. However, the water soluble sulfo-derivative of 8-HQ 5-(*p*-sulfophenylazo)-8-hydroxyquinoline showed higher acid dissociation constants, *pKa*_1_ of 3.78 and *pKa*_2_ of 7.94 [32]. These values would certainly change when the ligand is bound on silica. Equilibrium constants for 5Ph8HQ silica binding to metals have been determined. The stability constant of Cu^2+^ with the grafted material was *K*° = 4.8 × 10^8^ [31], showing higher stability constant with Sulfo-5Ph8HQ in water, *K* = 1.4 × 10^10^ [32]. Recent study has showed higher equilibrium constant for Cu^2+^ than the previously reported ones for 5Ph8HQ in micelles and colloidal fumed silica-5Ph8HQ, *K*° = 1.27 × 10^11^ and *K*° = 7 × 10^11^, respectively [33]. Stability constant for Ni^2+^ was also determined and found to be *K*'ext = 5.9 [24] and *K*'ext = 0.8–1.2 for 1 : 1 ligand/metal stoichiometry in presence of either acetate or chloride counter ion [33]. Stability constants of metals with 8-HQ immobilized silica according to Luhrmann et al. [28] protocol were also determined, which involved the functionalization of p-aminopropyl silica with 5-chloro-methyl-8-hydroxyquinoline. Using Lührmann protocol, distribution ratios log⁡⁡*K*_*D*_ were determined for many metals including Cu, Ni, Co, Fe, Mn, Cr, Zn, Cd, Hg, and Pb using batch equilibrium technique for 8-HQ-grafted silica. The use of the former protocol is not widely studied because of its extremely high cost. Many studies have used 5Ph8HQ for the retention and preconcentration of metals with excellent recoveries [5, 34–38]. Nevertheless, the adsorption dynamics of 5Ph8HQ-grafted silica is not completely studied particularly for mercury. Thus, in this study we attempt to study the adsorption kinetics of mercury in single- and multielement solution to discover the competition demonstrated between mercury and different metals for 5Ph8HQ affinity sites. Moreover, in this study, adsorption isotherms of mercury and other metals were determined for 5Ph8HQ. Finally, an analytical method for the determination of Hg(II) in river water was developed based on 5Ph8HQ-linked silica as adsorbent and Cold Vapor Atomic Fluorescence Spectroscopy (CV-AFS) as an analytical instrument.

## 2. Materials and Methods

### 2.1. Chemicals and Materials

#### 2.1.1. Reagents and Apparatus

Analytical reagent-grade chemicals were used and obtained Sigma Aldrich (France). Ultrapure water (with a resistivity of 18.2 MΩ) was obtained using a Milli-Q system (Millipore, USA). Silica (100–200 mesh), chemicals, and solvents were supplied from Sigma Aldrich (France). Labware was soaked in 20% nitric acid and washed thoroughly with pure water prior to use. Working solutions of metals ions (Cr(III), Cu(II), Co(II), Zn(II), Ni(II), Pb(II), Cd(II), As(II), Fe(III), V(II), and Hg(II)) were prepared from stock metal nitrate solution (1000 mg/L) (Merck). Buffer solutions were prepared from 1 M sodium acetate to which different volumes of 1 M nitric acid were added to obtain pH in the range 4–6. Ammonium acetate and phosphate buffer were used for obtaining pH 7 and pH 8, respectively.

Fourier transform infrared (FT-IR) spectra of functionalized silica were recorded using Nicolet 380 Smart *i*TR spectrometer (ThermoScientific, USA) and were silica-matrix corrected. An inductively coupled plasma mass spectrometer, Thermo-Optek X7 (Thermo Fischer Scientific, USA), equipped with a cross-flow pneumatic nebulizer, a concentric glass-type nebulization chamber and a 1.5 mm i.d. quartz plasma torch, was used. All the operating parameters were those recommended by the manufacturer. The optimum operating conditions and measurement parameters for ICP-MS are listed in Table 1. Measurements were performed with high-purity Argon gas. The pH measurements and adjustments were conducted by pH meter (Thermo Scientific Orion Star A111, USA). The flow rates of the samples were adjusted using a Gilson Miniplus 3 peristaltic pump. PVC tubes (3.18 i.d) were used for the preconcentration process. Self-made PTFE (polytetrafluoroethylene) columns (65 mm × 4 mm i.d.) were used for packing the examined adsorbent. Mercury was measured by cold vapor atomic fluorescence spectroscopy (Tekran, Model 2600 CVAFS Mercury Analysis System, USA). Ionic mercury is reduced with SnCl_2_ (2% solution in 1% HCl), subsequently converted from Hg(II) to Hg°. The volatile species of mercury are separated from solution by purging with high-purity argon gas through a semipermeable dryer tube. Volatile mercury is then carried by argon gas and preconcentrated into a gold cell of the cold vapor atomic fluorescence spectrometer. After thermal desorption, the concentration of Hg is determined by atomic fluorescence spectrometry at 253.7 nm.

### 2.2. Synthesis of 5Ph8HQ-Linked Silica Gel

Phenylazo-8-hydroxyquinoline silica gel was prepared according to the procedure proposed by Sugawara et al. [34]. The procedure firstly consists in a coupling step between an arylamine moiety and aminopropyl silica gel (APSG) to permit further azo-linking of 8-hydroxyquinoline on position 5. APSG (10 g) was also acylated by treatment with *p*-nitrobenzoyl chloride (1 g) in chloroform (60 mL) in presence of triethylamine (2 mL). The nitro adduct was then reduced by sodium dithionite (5% w/v solution) to yield aminobenzoyl-APSG. Oxidation of the amino moiety with sodium nitrite afforded formation of the expected diazonium salt, which was rapidly filtered to be coupled with 8-HQ. Formation of the final adduct was characterized by the rapid development of a deep red color, prominent feature of the formation of the expected diazo-linkage. Presence of the anchored 8-hydroxyquinoline was further ensured by FT-IR analysis, which revealed absorption bands from 1636 to 1524 cm^−1^ relative to the presence of amide and heteroaromatic bonds.

### 2.3. Adsorption Experiments

A series of metal sorption experiments were conducted to study the effect of pH, sorption kinetics, selectivity, adsorption capacities, and interferences of mercury and some other metals. Batch experiments were conducted using 1000 mL polyethylene (PE) bottles. The amount of adsorbent used was 1 g and the volume of ultrapure water was maintained at 200 mL. A certain concentration of metal ion was prepared by dilution of stock solution (1000 mg/L M^+^) and adjusted to the required pH using an adequate buffer system. The bottles were shaken at room temperature for a fixed period of time at constant speed 150 rpm. At the end of shaking time, the supernatant was separated from the solid phase by filtration using Millipore filters and analyzed by ICP-MS for metals, and mercury analysis was performed using CV-AFS. The experiments were conducted in duplicates and the average results were reported. This methodology was followed to identify the pH effect, optimize shaking time, and understand the competition between metals towards 5Ph8HQ sites and its effect on mercury adsorption efficiency.

Metal uptake or sorption capacity was calculated based on the difference of metal ion concentration before and after adsorption according to (1). The percentage of mercury sorption, distribution coefficient, selectivity coefficients, and half-life sorption [39] were calculated using (1)–(5), respectively, as follows:

(1) $$\begin{array}{c} \text{Sorption}\left(\%\right)=\frac{C_{i}-C_{\text{eq}}}{C_{i}}\times 100, \end{array}$$

(2) $$\begin{array}{c} Q=\frac{\left(C_{i}-C_{\text{eq}}\right)V}{m}, \end{array}$$

(3) $$\begin{array}{c} K_{d}=\frac{Q}{C_{\text{eq}}}, \end{array}$$

(4) $$\begin{array}{c} \alpha =\frac{K_{d}\left(\text{Hg}\left(\text{II}\right)\right)}{K_{d}\left(X\right)}, \end{array}$$

(5) $$\begin{array}{c} t^{1/2}=\frac{1}{K_{2}Q_{\text{eq}}}. \end{array}$$

Here, *C*_*i*_  (mg/L) is the initial concentration; *C*_eq_ (mg/L) is the equilibrium concentration in the solution; *V* (L) is the solution volume; *m* (g) is the amount of sorbent; *Q* (mg/g) represents the sorption capacity and *Q*_eq_ represents the sorption capacity at equilibrium; *K*_*d*_ is distribution coefficient; *α* is the selectivity coefficient for the binding of a specific metal in the presence of other competitive ions; *X* represents the metal ion species.

### 2.4. Kinetics Studies

A certain concentration of metal ion solution was prepared and adjusted to the optimized pH using buffer system. An accurately measured sorbent of 1 g was added. Different aliquots were sampled and filtered at different time intervals for 24 hours. Sorption dynamics were studied in mercury single metal solution and binary solutions of Hg(II)-Cu(II), Hg(II)-Co(II), Hg(II)-Ni(II), Hg(II)-V(II), and Hg(II)-Fe(III). Moreover, adsorption kinetics were performed in multielement mixture composed of Hg(II), Cr(II), Fe(III), V(II), As(II), Co(II), Cu(II), Ni(II), Cd(II), Pb(II), and Mn(II).

Sorption kinetics were analyzed using pseudo-first- and second-order rate equations and intraparticle diffusion model. Chemical reactions in homogenous systems are described as pseudo-first-order rate equation. The linearized form of the first-order rate equation by Lagergren [40] is given as follows:

(6) $$\begin{array}{c} \text{Log}\;\left(Q_{\text{eq}}-Q_{t}\right)=\log Q_{\text{eq}}-\frac{K_{1}t}{\text{2,303}}, \end{array}$$

where *Q*_eq_ and *Q*_*t*_ are the amounts of metal ions adsorbed at equilibrium (mmol/g) and at contact *t* (min), respectively, and *K*_1_ (1/min) is the rate constant.

The linearized equation of pseudosecond order rate equation was given by Ho and McKay [41] as follows:

(7) $$\begin{array}{c} \frac{1}{Q_{t}}=\frac{1}{K_{2}Q_{\text{eq}}^{2}}+\left(\frac{1}{Q_{\text{eq}}}\right)t, \end{array}$$

where *K*_2_ (g/mmol·min) is the rate constant of pseudo-second-order adsorption reaction.

Intraparticular diffusion can be described to the model given by [42] by the following:

(8) $$\begin{array}{c} Q_{t}=K_{i}t^{1/2}, \end{array}$$

where *K*_*i*_ is the intraparticle diffusion rate constant (mmol/g·min^0.5^).

### 2.5. Adsorption Isotherms

Adsorption isotherms were determined for Hg(II). Accurately measured 1 g of the sorbent was mixed with 50 mL of solution adjusted to the Hg optimal pH. The concentration of metal ion was changed between 10–160 mg/L while being shaked (agitation speed 150 r/min). The contact time was selected on the basis of the kinetics studies. At equilibrium time, an aliquot was sampled, filtered, and analyzed. The results were adjusted to the Langmuir, Freundlich, and Dubinin-Radushkevich (D-R) models [43–45]. The Langmuir model linearized form is given by the following:

(9) $$\begin{array}{c} \frac{1}{Q_{\text{eq}}}=\frac{1}{Q_{m}}+\frac{1}{bQ_{m}C_{\text{eq}}}, \end{array}$$

where *Q*_eq_ (mmole/g) is the amount of metal ion adsorbed, *C*_eq_ is the equilibrium metal ion concentration (mmol/L), *Q*_*m*_ (mmole/g) is the maximum Langmuir uptake when the surface of the adsorbent is completely covered with adsorbate, and *b* (L/mmole) is the Langmuir adsorption constant.

The Freundlich linearized form is described by (10):

(10) $$\begin{array}{c} \text{Log}\;Q_{\text{eq}}=\log K_{F}+\frac{1}{n\log C_{\text{eq}}}, \end{array}$$

where *Q*_eq_ is the equilibrium metal ion concentration on the adsorbent (mmol/g), *C*_eq_ is the equilibrium concentration of metal ion (mmole/L), *K*_*F*_ is the Freundlich constant (mmole/g) which indicates the adsorption capacity and the strength of the adsorption, and *n* is the heterogeneity factor representing bond distribution.

The D-R adsorption isotherm linearized form is given by the following:

(11) $$\begin{array}{c} \ln Q=\ln Q_{m}-K\varepsilon^{2}, \end{array}$$

where *Q* is the amount of metal ion adsorbed per unit weight of the sorbent (mole/g), *K* is a constant related to the adsorption energy (mol^2^/KJ^−2^), *Q*_*m*_ is the maximum adsorption capacity (mole/g), and *ε* is the Polanyi potential (J/mole).

### 2.6. Preconcentration Study

For optimization procedure, the synthesized 5Ph8HQ was cleaned by 20 mL of 2 M HNO_3_ at flow rate 2 mL/min, washed by Milli-Q pure water until free from acid, and finally conditioned by acetate-acetic acid buffer system at flow rate 1 mL/min. Aliquots of 50 mL of river water containing of 2 ng mL^−1^ were adjusted to optimal extraction pH using ammonia or HNO_3_ Suprapur solution. Samples were percolated through 300 mg precleaned resin at optimized flow rate, eluted with a suitable eluent, and finally analyzed by CV-AFS.

## 3. Results and Discussion

### 3.1. Effect of pH

The effect of pH variation on metal uptake of Hg(II), Cr(II), V(II), As(II), Co(II), Cu(II), Ni(II), Cd(II), Pb(II), Mn(II), and Zn(II) was investigated using batch procedure in single-metal solution. Solution acidity was varied between pH 3 and 8. At equilibrium time, aliquots were sampled, filtered, and analyzed. Metal ion sorption can occur through several single or mixed mechanisms. Coordination with the functional groups adhered to the sorbent through chelation, electrostatic attraction, and anion exchange with protonated amino group through proton or anion exchange.

**Figure 1:**
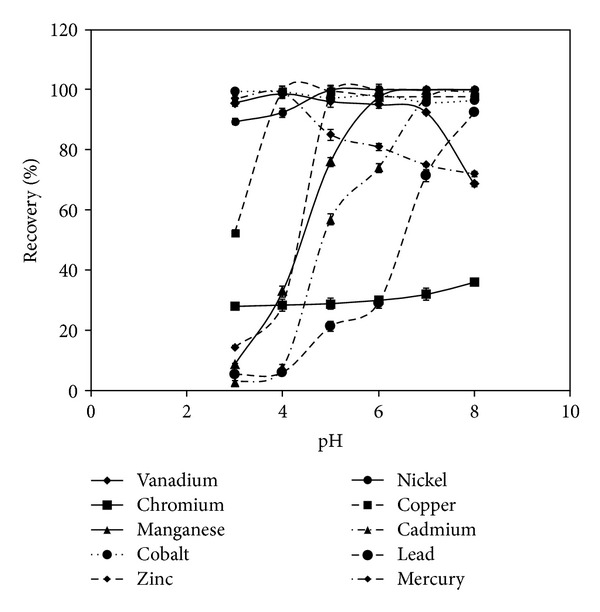
Effect of pH variation on the adsorption of metals. Conditions: volume: 200 mL, m(5Ph8HQ): 1 g, and [M^+^]: 1 *μ*g mL^−1^ in single-metal solution. Bars represent standard deviation for two replicates.

Figure 1 shows the effect of pH variation on metal retention on the grafted material. The optimal adsorption of mercury was found in the range 3-4 with pH 4 as the optimal acidity. Above pH 4, Hg(II) retention decreases to 80% and follows a steady trend between pH 4 and 8. At pH greater than 5, mercury forms colloidal precipitate of Hg(OH)_2_ or soluble Hg(OH)^+^. The chemistry of mercury in aqueous solutions is more complicated than any other metal. Mercuric ions can be easily hydrolyzed according to the equation [46]. The coordination of water influences mercury adsorption dynamics and can relatively allow for slow diffusion,

(12) $$\begin{array}{c} {\text{Hg}{\left({\text{H}}_{2}\text{O}\right)}_{6}}^{2+}\to {\left[\text{Hg}{\left({\text{H}}_{2}\text{O}\right)}_{5}\text{OH}\right]}^{+}+{\text{H}}^{+}. \end{array}$$

The presence of Cl^−^ can result in the formation of HgCl^+^, HgCl_2_ and HgCl_4_^2−^, HgCl_3_^−^ [47] and in the possible electrostatic adsorption on the resin depending on its charged form. The increase in retention might be attributed to these species and not due to free Hg(II). Lower pH values are expected to enhance protonated ligand sites available for adsorption. Beyond pH 6, mercury uptake can decrease due to precipitation for high concentration, which is therefore not applicable in our case. The pH dependence of metal adsorption is the result of the effect of metal speciation and the ionization forms of different groups of the sorbent. The adsorption of mercury is possibly complexation and not chemical binding since the zeta potential of 5Ph8HQ is zero [33]. The affinity of mercury toward the donor set atoms of 5Ph8HQ is due to hard and soft acid base theory. It correlates the degree of metal softness to the observed strength of interaction with the donor atoms (N, O). Mercury is considered as a soft metal, because of its relatively large ionic size, low electronegativity, and high polarisability. The N=N, –NH, and OH of 5Ph8HQ are considered relatively soft bases leading to the observed high affinities between each other.

The stability constant of Cu(II) with 8-HQ is elevated (log⁡*β* = 12.56) [48], which is demonstrated by high distribution coefficient value as discussed below. Quantitative adsorption of Cu(II) is found for pH values greater than 4 and then slightly decreases due to the formation of strong complexes with carbonates Cu(CO_3_)_2_^2−^ (log⁡*β* = 10.69) [49]. The retention of Cd(II) is shown to be low for pH < 6, in which Turner et al. [49] reported that 96% of Cd are found as ionic free species for pH = 6. In seawater, it has been reported that when pH < 6, Cd forms chlorocomplexes CdCl_2_, CdCl_3_^−^, and CdCl_4_^−^ with high stability constants log⁡*β* = 2.59, log⁡*β* = 2.4, and log⁡*β* = 1.47 [49], respectively, which impedes its complexation with 8-HQ. Thereby, quantitative retention is achieved for pH values >6, with the formation of weak hydroxide complexes Cd(OH)^+^, Cd(OH)_2_, and Cd(OH)_3_^−^ of log⁡*β* = −10.08, log⁡*β* = −20.35, and log⁡*β* = −33.3 [49]. The stability constant of Cd with 8-HQ is high, log⁡*β* = 7.78, which can easily break down the weak complexes formed with hydroxides. As for Mn(II), quantitative retention is attained for pH values >6. At basic medium, Mn forms weak complexes with hydroxides Mn(OH)^+^ (log⁡*β* = −10.59), carbonates Mn(CO_3_) (log⁡*β* = 4.1) [49] which seems far from the competition with 8-HQ complexes (log⁡*β* = 6.8).

At acidic pH values, the stability constant of Ni(II) (log⁡*β* = 9.27) and H^+^ (log⁡*β* = 9.9) with 8-HQ is comparable. Therefore, quantitative recoveries of Ni(II) are found for pH ≥ 5 values although at pH 4 92.4% recovery of Ni(II) can be attained. As shown in Figure 1, the retention of Pb(II) at low pH values is almost negligible. This is due to the competition between Pb(II) and H^+^ to form stable complexes with 8-HQ with log⁡*β* = 9.1 and log⁡*β* = 9.9, respectively. As have been described for Ni(II) and Pb(II), Zn(II) follows the same behavior, with competition of H^+^ ions at low pH values (log⁡*β* = 9.9) to form more stable complexes with 8-HQ than Zn-8-HQ complexes (log⁡*β* = 8.56). Therefore, quantitative retention of Zn(II) is observed for pH ≥ 5.

Quantitative recovery of Vanadium is only seen for 3 < PH < 4. At higher pH values, the retention decreases. This is explained by the formation of V(IV) species at pH values <3.5 which are mainly VO^2+^, VO(OH)^+^, (VO(OH))_2_^2+^, and VO(OH)_2_ [50]_._ At 3.5 < pH < 7.5, anionic species form, mainly H_2_VO_4_^2−^, and for 7.5 < pH < 13, the anionic form of HVO_4_^2−^ dominates [51]. The retention of Cr(III) at the studied pH range (3–8) is lower than 36%. It is probably due to the formation of chromium, under the oxidation state (V), which forms CrO_4_^2−^ which certainly obstructs its complexation with 8-HQ [52].

Arsenic showed no adsorption by 5ph8HQ at the studied pH range (pH 3–8). In summary, the adsorption of Pb(II) Cd(II), and Mn(II) was almost negligible between pH 3 and 4, then it increased at pH > 5. It is shown that basic conditions are favorable for Pb(II) retention. At lower pH value, the retention of these metals drops off probably due to the partial protonation of active sites and the stronger electrostatic repulsion between Pb(II) and the protonated amino and hydroxyl groups of 5Ph8HQ. At basic medium, the retention is higher which is appreciated in selective separation from Hg(II), V(II), Cu(II), Ni(II), and Co(II). Moreover, at basic pH values, functional groups of the sorbent are free from protonation, and therefore, retention is controlled by chelation mechanism. The corresponding affinity of the resin is attributed to the protonation of the free lone pair of nitrogen suitable for coordination with metals. Values of pH higher than 8 were not examined to prevent hydrolysis of adsorbent and precipitation of metals. The optimal pH values for Hg(II) is 4, for V(II) is 4, for Cu(II) is 4, for Co(II) is 3-4, for Zn(II) and Ni(II) is 5–8, for Cd(II) and Pb(II) is 8, for Cr(II) is 7, for Mn(II) is 7–8. Therefore, at pH 4, simultaneous extractions Hg(II), V(II), Cu(II), Co(II), and Ni(II) can be achieved by 5Ph8HQ with maximal extractions occurring for Hg(II), V(IV), Cu(II), and Co(II) with prominent separation from Pb(II), Cd(II), Mn(II), and Zn(II).

### 3.2. Adsorption Dynamics

**Figure 2:**
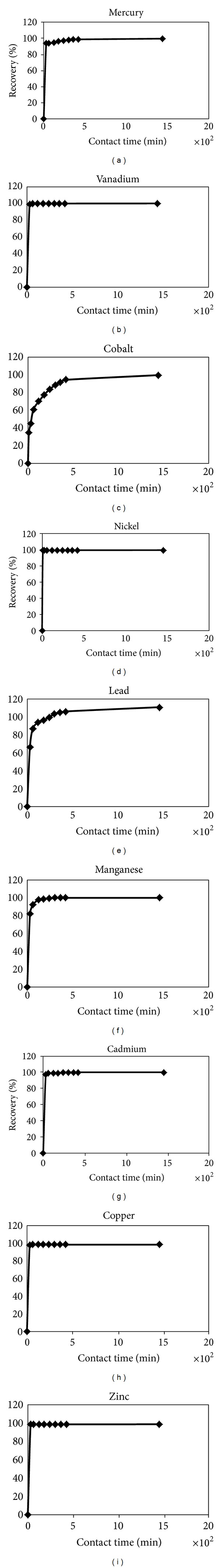
Recovery percentage of metals as function of time in a single-metal solution. Conditions: volume: 200 mL, m(5Ph8HQ): 1 g, element optimized pH, and [M^+^]: 1 *μ*g mL^−1^.

In order to determine the optimum contact time between 5Ph8HQ, adsorption recoveries (%) were measured as a function of time in single-metal solution and presented in Figure 2. Rapid uptake of metals by the resin was observed within the first 10 minutes. The time required to reach adsorption equilibrium was 30 minutes for V(IV), Cd(II), Cu(II), Zn(II), Hg(II), Ni(II), while slower equilibrium time was attained for Mn(II) 1 hour, and 5 hours for Co(II) and Pb(II). Adsorption time equilibrium was comparable to other resins, and in some cases, the adsorption is faster than others reported in the literature [53, 54]. On the other hand, in multielement mixture, equilibrium time of Hg and other metals has shifted to longer time, mainly because of interferences with other metals with stronger affinity to 5Ph8HQ. Mercury has reached equilibrium after 2 hours, while for Co(II), Ni(II), Zn(II), and Fe(III), the adsorption time was longer and did not reach quantitative recoveries after 24 hour contact time. Still, the equilibrium time of Cu(II) and V(IV) has not been influenced in multielement mixture, revealing the significant affinity of these metals towards the studied resin.

In a single metal solution, the lowest half-life sorptions were that of Cu(II) 0.06 min < V(IV) 0.28 min < Ni(II) 0.39 min < Zn(II) 0.41 min < Cd(II) 0.89 min, respectively, while slower sorption kinetics were found for Hg(II) 5.39 min < Pb 25.23 min < Co(II) 49.96 min (Table 2). Overall, the fast adsorption of metals on 5Ph8HQ can be attributed to the good hydrophilicity of silica and the chelating strength of 5Ph8HQ towards metals. In order to interpret the sorption kinetics, Lagergren first-order equation, pseudo-second order equation, and Webber-Morris intraparticular diffusion models were used to evaluate the experimental data.

**Figure 3:**
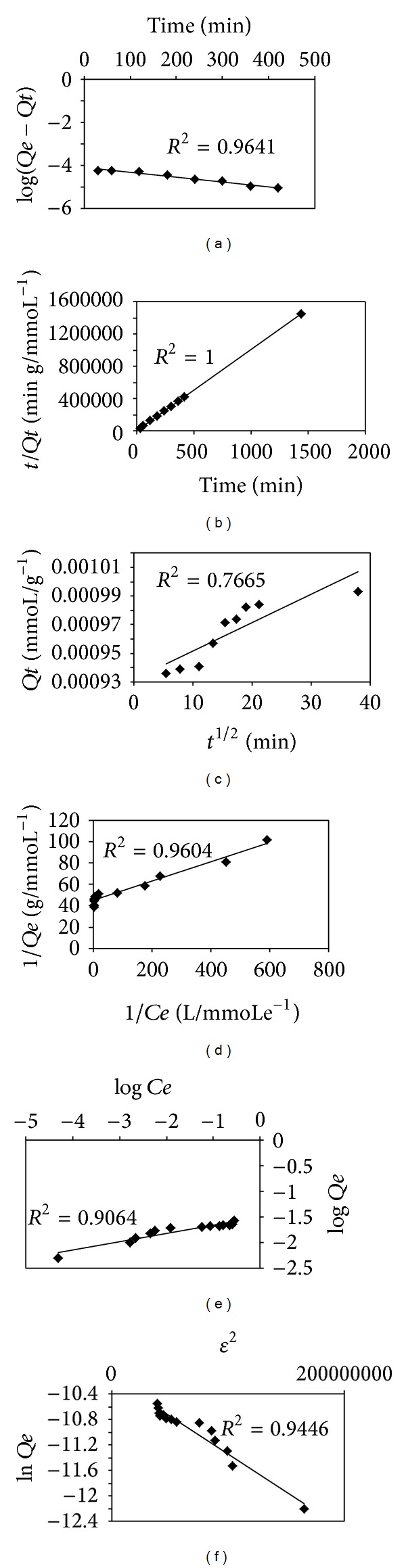
Adsorption kinetics of Hg(II) according to (a) pseudo-first-order model, (b) pseudo-second-order model, (c) intraparticular diffusion; [Hg^2+^]: 0.005 mmol L^−1^, volume: 200 mL, pH: 4, and m(5Ph8HQ): 1 g. Adsorption isotherms of Hg(II) by 5Ph8HQ according to (d) Langmuir adsorption model, (e) Freundlich adsorption model, and (f) Dubinin-Radushkevich adsorption model; [Hg^2+^]: 10–160 *μ*g mL^−1^, volume: 50 mL, pH: 4, and m(5Ph8HQ): 1 g.

Table 2 shows adsorption kinetic parameters of different metals in single-metal solution. First-order rate *K*_1_ was determined from the slope and intercept of the plot of log⁡(*Q*_eq_ − *Q*_*t*_) versus *t*. The correlation coefficients (*R* ^2^) were between 0.99–0.79 for all studied metals and 0.9641 for Hg(II). As for pseudo second-order model, correlation coefficients were superior to 0.999 for all systems. The inapplicability of the first kinetic model suggests the prevalence of pseudo-second-order kinetic model on the adsorption of metals on the studied sorbent in which the calculated equilibrium adsorption capacities *Q*_*e*_ (theoretical) were in accordance with the experimental adsorption capacities *Q*_*e*_ (experimental). Thereby, chemisorption may control the adsorption mechanism involving valence forces through sharing or exchange of electrons between the resin and metals [55]. Moreover, the kinetic results were also fitted to intraparticular diffusion model by tracing *q*_*t*_ versus *t* ^1/2^. If the plots were straight lines passing through the origin, then the adsorption mechanism is governed by intraparticular diffusion defining the sole rate limiting step. If plot *q*_*t*_ versus *t* ^1/2^ did not pass the origin, then the adsorption might be dominated by film diffusion or boundary layer diffusion [56]. As shown in Figure 3, the corresponding plot was not linear over the studied time range. Hg adsorption had an initial linear portion followed by a plateau suggesting the governance of intraparticular diffusion in the few hours of adsorption then film diffusion dominated. These two different stages of adsorption for Hg(II) are also seen for Co(II), V(IV), Cd(II), Cu(II), Zn(II), Ni(II), and Pb(II) with good correlation coefficients attained for Zn of 0.8 and Co, Ni, and Hg of 0.76. In contrast to other metals, Mn(II) demonstrated only film diffusion where a plateau is only seen.

In order to examine the influence of metals on the adsorption kinetics of mercury, adsorption kinetics were examined in multielement mixture. For this purpose, a certain metal ion concentration containing V(IV), Cr(II), Co(II), Ni(II), Cu(II), Zn(II), Fe(III), and Hg(II) was added to 1 g of 5Ph8HQ at optimal pH for mercury sorption (pH 4). Metal mixture was shaken at 150 r.p.m and samples were taken at predetermined time as described above. As demonstrated in Table 3, the experimental data fitted-pseudo-second-order model with correlation coefficient between 0.9 and 1. The calculated equilibrium adsorption capacities for almost all metals tested were consistent with experimental data except for Co(II), Ni(II), and Zn(II). It seems that the adsorption of Ni, Co, and Zn is significantly affected in multielement mixture at pH 4. Similar to single-metal solution, film diffusion predominated the trend of adsorption of metals in multielement mixture except for Co which followed intraparticular diffusion model with correlation coefficients of 0.97. Higher half-life sorption was found for all metals in multielement mixture, in which *t* ^1/2^ of some metals was tremendously affected. The *t* ^1/2^ of Ni(II) and Zn(II) were 230 and 61 higher in magnitude, respectively, than in single-metal solution. The sorption sequence of metals has been altered in multi-element solution with fast adsorption for Cu, V, and Hg. The *t* ^1/2^ sorption sequence became as follows: Cu(II) 0.7 min < V(IV) 1.86 min < Hg(II) 14.52 min < Zn(II) 25 min < Ni(II) 89.83 min < Fe(III) 124 min < Co(II) 119.61 min (Table 3). Therefore, in multi-element solution, Cu(II) and V(IV) appear to compete with Hg(II) for the adsorption sites of 5Ph8HQ since these metals have faster sorption times.

### 3.3. Competitive Adsorption

#### 3.3.1. Multi-Element Mixture

The selectivity is determined by equilibrating a unit mass of the adsorbent in solution containing the same initial concentration for all metals. Solution acidity was adjusted to 4, which is the optimal pH for mercury adsorption. The overall adsorption efficiencies of Cu(II), V(IV), and Hg(II) remained unaffected in multi-component mixture. Maximum metal retention of 99.9%, 99.8%, 99.3%, 94.2%, 81%, 79%, 32.5%, 14%, and 10% were obtained for Cu(II), Hg(II), V(IV), Fe(III), Co(II), Ni(II), Zn(II), Pb(II), and Cd(II), respectively. As a result, the affinity order can be as follows: Cu(II) > Hg(II) > V(II) > Fe(III) > Ni(II) > Co(II) > Zn(II) > Pb(II) > Cd(II). The highest affinities (*K*_*d*_) were obtained for Cu(II) 153.64, Hg(II) 50, and V(IV) 25 (Table 4). According to separation factor (*α*), mercury can be separated from metals of high affinity to 5Ph8HQ, that is, V(IV), Fe(III), Co(II), and Ni(II); however, Cu(II) poses a potential concurrence with Hg(II) towards the resin binding sites. This is ascribed to the higher distribution coefficient *K*_*d*_ Cu(II) 145.48 > *K*_*d*_ Hg(II) 50 and *α* < 1.

#### 3.3.2. Binary and Single Metal-Solution

Following the results of multi-element mixture, the highest affinities of metals towards 5Ph8HQ are obtained with an order: Cu(II) > Hg(II) > V(IV) > Fe(III) > Ni(II) > Co(II). Therefore, it is interesting to identify the effects of these metal ions with mercury in binary metal mixture at pH 4. The outlined order of metal ion selectivity by 5Ph8HQ based on the values of distribution coefficient and separation factor *α* is Cu(II) > Hg(II) > Co(II) > V(IV) > Ni(II) > Fe(III) (Table 5). It seems that the affinity order as seen in Table 4 has changed in binary metal mixture as compared to multi-element mixture with increased values of  *K*_*d*_ and a decrease in the separation factor *α*_kHg/kMm+_. Change in the affinity of metals towards different sorbents with varying the competition potential was also observed in previous adsorption studies [57, 58]. According to the Irving-Williams series, the stability of metal complexes with ligands is in the order: Mn(II) < Fe(II) < Co(II) < Ni(II) < Cu(II) > Zn(II) [59]. The crystal field theory suggests electrostatic interaction between the central atom and the ligand. Therefore, there is a direct relationship between stability of metal complexes and ionic potential that is the charge to radius ratio (*Z/r*). Following the *Z/r* ratio cited in Table 6 of the studied metals, the selectivity sequence is expected to be Mn(II) > Ni(II) > Co(II) = Zn(II) > V(II) > Cd(II) > Hg(II) and for trivalent metals As(III) > Cr(III) > Fe(III). The stability sequence of metals is not consistent with our results in either of the two mixed metal studies. The adsorption selectivity can shift from the predicted affinities depending on the experimental conditions (pH etc.) and sorbent properties. A direct correlation is made between *Z/r* ratio of metals and the metal complex stability in pure cation-exchange mechanism [60] that could be not ascribed to the studied resin. Therefore, it is also important to define the selectivity and the stability constants in single-metal solution without the presence of competitor elements.

Distribution coefficients are determined in single-metal solution at element-optimized pH. As demonstrated by Table 7, *K*_*d*_ has tremendously been altered to high values as opposed to that observed in multi-element mixture; still, *K*_*d*_ values for Cu(IV), V(II), and Hg(II) remained unchanged. Therefore, according to *K*_*d*_ values, metal affinity order towards 5Ph8HQ can be as follows: Mn(II) 999.8> Co(II) 799.8 > Cd(II) 199.8 > Cu(II) 160 > V(IV) 137.73 > Ni(II) 59.5 > Hg(II) 52.37 > Zn(II) 18.84 > Pb(II) 2.4.

### 3.4. Adsorption Isotherms

**Figure 4:**
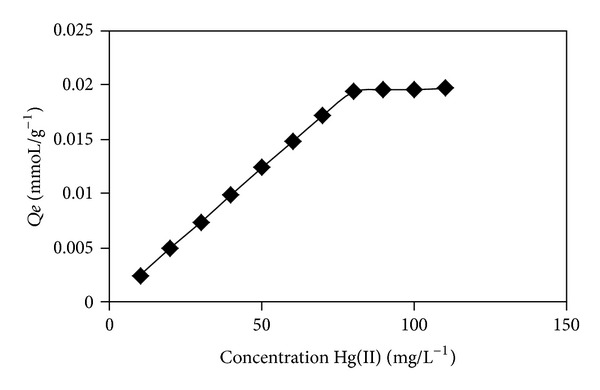
Adsorption profile of Hg(II) as a function of increasing metal concentration. Conditions: [Hg^2+^]: 10–160 *μ*g mL^−1^, volume: 50 mL, pH: 4, and m(5Ph8HQ): 1 g.

Adsorption isotherm studies are of great value for their importance in describing the interaction between the solute, adsorbent, and the adsorbate. Thereby, the effect of initial concentration of metal sorption was investigated by varying the initial concentrations of metal ions at optimum pH. The obtained results were presented in Figure 3. As indicated in Figure 4, as the concentration of metal ion increases, the larger is the equilibrium adsorption uptake by the adsorbent. There seems to be a direct relation between the loading capacity of the sorbent and the metal ion concentration. It can be explained by the fact that, with higher concentration, the transfer driving force is larger [61]. The adsorption data for metals were analyzed and fitted according to Langmuir, Freundlich, and Dubinin-Radushkevich (D-R) models. The Langmuir isotherm model suggests monolayer sorption at specific homogenous site without interaction between the sorbed molecules, with sites having identical adsorption energies. The Freundlich isotherm sorption assumes a monolayer sorption on heterogeneous sites with interaction between the adsorbent and that the adsorbate and all adsorption sites are energetically different. The D-R isotherm is considered more general than Langmuir model; it does not assume homogenous surface or constant adsorption potential. It is used to identify whether the adsorption is of a chemical nature or a physical one [44].

The model constants of Langmuir, Freundlich, and D-R isotherms along with correlation coefficients values are listed in Table 8. The *R* ^2^ values indicate that Langmuir fit the experimental data better than Freundlich and D-R isotherm models, suggesting a monolayer coverage of Hg(II) on 5Ph8HQ resin. Langmuir isotherm adsorption type was also found for Hg(II) with other studied resins, thiourea-modified chitosan [62] and poly-allyl thiourea [13]. The maximum adsorption capacity of Hg(II) on 5ph8HQ was found to be 0.022 mmole Hg(II)/g corresponding to a maximum Hg(II) initial concentration of 90 mg/L (Figure 4). The determined adsorption capacity is considered as minimal as compared to other resins such as thiol-functionalized mesoporous silica magnetite nanoparticles with 0.8 mmole Hg(II)/g [63] and 0.39 mmole Hg(II)/g of silica supported dithiocarbamate [64], 1.13 mmole Hg(II)/g of poly-allyl thiourea [13]. 8-Hydroxyquinoline silica synthesized according to Lührmann et al. [28] showed higher capacity for Hg(II) 0.448 mmole/g at optimized pH 6. The adsorption capacity for some metals has also been reported using HQ-silica synthesized according to different protocols. Marshall and Mottola [7] found an adsorption capacity for Cu(II) 0.227 mmole/g, while HQ-silica synthesized according to Pyell and Stork [27] showed higher capacity of 0.414 mmole/g and 0.248 mmole/g for Ni(II) and 0.333 mmole/g for Zn(II).

For Langmuir model, to determine if the resin is favorable for Hg(II) sorption, a dimensionless separation factor is defined as:

(13) $$\begin{array}{c} R_{L}=\frac{1}{1+K_{L}C_{i}}, \end{array}$$

where *K*_*L*_ (L/mmole) is the Langmuir equilibrium constant and *C*_*o*_ (mmole/L) is the initial concentration of metal ion. If *R*_*L*_ > 1, the isotherm is unfavorable; if *R*_*L*_ = 1, the isotherm is linear; if 0 < *R*_*L*_ < 1, the isotherm is favorable; if *R*_*L*_ = 0 the isotherm is irreversible [65]. The value of *R*_*L*_ in this study was found to be 0.01, confirming the suitability of the resins for the recovery of Hg(II) ions. Moreover, the low *R*_*L*_ value <0.1 implies strong interaction between Hg(II) and 5Ph8HQ.

### 3.5. 5Ph8HQ Column Extraction of Hg(II)

#### 3.5.1. Flow Rate Optimization

**Figure 5:**
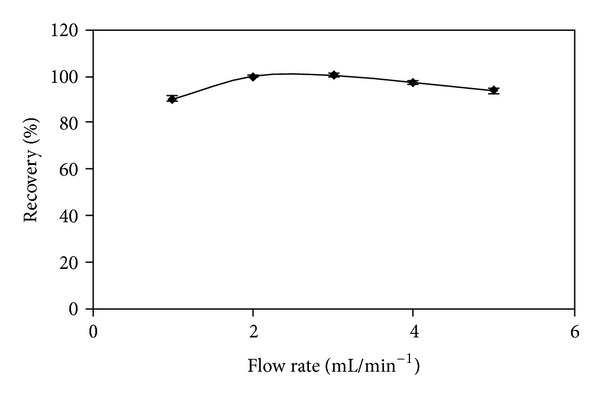
Effect of flow rate mercury by 5Ph8HQ column extraction. Conditions: [Hg^2+^]: 2 ng mL^−1^, *V* (50 mL), pH 4, and eluent: 5 M HCl + 5% CS(NH_2_)_2_/10 mL. Bars represent standard deviation for two replicates.

The flow rate of Hg(II) solution through the column is an important parameter, in order to ensure sufficient contact time between the sorbent and the adsorbate and to control time of analysis. The flow rate of samples was investigated in the range between 1 and 5 mL/min. As shown in Figure 5, quantitative recoveries were obtained for flow rates between 2 and 3 mL/min. At flow rate > 3 mL/min, recoveries drop off to 93%. Therefore, a flow rate of 2 mL/min was used for the subsequent Hg(II) preconcentration.

#### 3.5.2. Eluent Type and Volume Optimization

**Figure 6:**
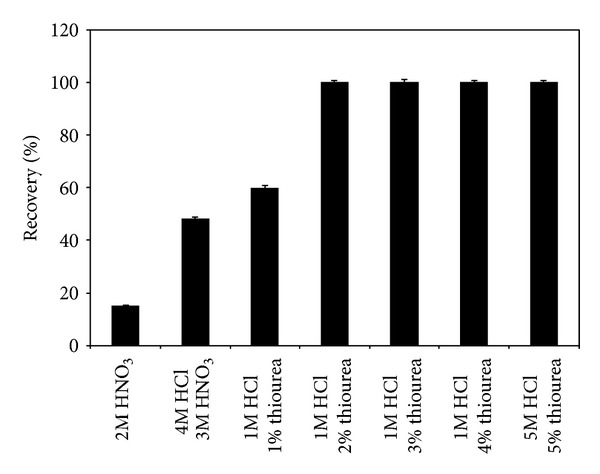
Elution recovery of Hg(II) adsorbed on 5Ph8HQ by column method using different types of eluents. Conditions: [Hg^2+^]: 2 ng mL^−1^, volume: 50 mL, pH: 4, and eluent volume: 10 mL. Bars represent standard deviation for two replicates.

**Figure 7:**
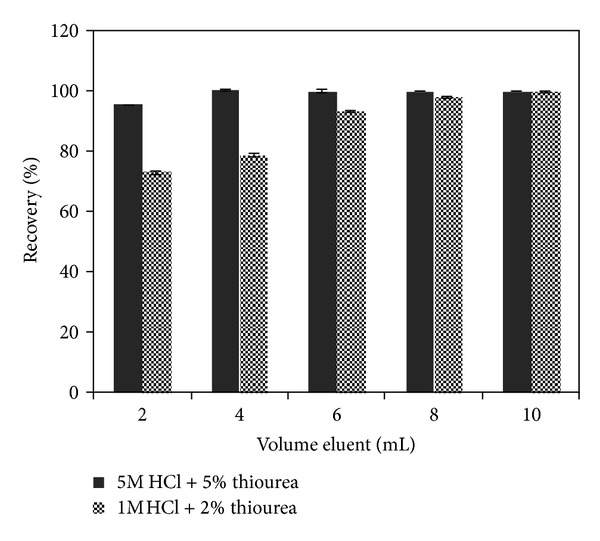
Effect of eluent volume on the recovery of Hg(II) adsorbed on 5Ph8HQ by column method. Conditions: [Hg^2+^]: 2 ng mL^−1^, *V* (50 mL), pH 4, and eluent: 5 M HCl + 5% CS (NH_2_)_2_/1 M HCl + 2% CS (NH_2_)_2_. Bars represent standard deviation for two replicates.

Elution of Hg(II) from 5Ph8HQ column was investigated by using different types of eluents, following the general procedure. The results demonstrated in Figure 6 showed that even with a mixture of HCl + HNO_3_, Hg(II) ions have not been completely desorbed. However, the use HCl + HNO_3_ as an eluent for metals from 5Ph8HQ was proven to be efficient [36, 37, 66]. This further accentuates the strong affinity of Hg(II) ions towards binding donor atoms (N, O) of 5Ph8HQ. In this case, as other studies have reported for the desorption of Hg(II) [13, 20], a strong complexing agent for mercury such as thiourea is added to varying concentrations of HCl. The obtained results show that 1 M HCl mixed with [CS(NH_2_)_2_] ≥2% was shown to be sufficient for quantitative desorption of Hg(II) ions (Figure 6). Even though 1 M HCl + 2% CS(NH_2_)_2_ was enough for the elution of Hg(II) ions, a larger elution volume of 10 mL was required for stripping off all the target ions. In order to achieve higher enrichment factor, 4 mL of 5 M HCl + 5% CS (NH_2_)_2_ was used for complete desorption at an optimized eluent flow rate of 0.5 mL/min (Figure 7).

#### 3.5.3. Interferences

As shown in mercury adsorption dynamics section, the coexistence of equimolar metals with Hg(II) can prolong the equilibrium time due to the competition between mercury and other metals towards the relevant binding sites. Therefore, the effect of common interfering ions on the adsorption of Hg(II) was examined using batch experiment. For that purpose, 1 g of the studied resin was equilibrated at pH 4 with 1 mg/L of Hg(II) and a certain concentration of the interfering ion. The mixture was shaken at 150 r.p.m and aliquots were taken at Hg equilibrium time and analyzed using CV-AFS. Results of Table 9 showed that several thousand fold excess of KNaC_4_H_4_O_6_, Na_2_CO_3_, Na_3_C_6_H_5_O_7_, humic acid had minor effect on the extraction of Hg(II) ions in which quantitative recoveries are still attained. Similarly, 10–100-fold excess of anions and major competing metals did not impose significant interferences, yet 4000-fold excess of Na_2_C_2_O_4_ and KCN have decreased the Hg(II) retention to 94% and 87%, respectively. The latter high concentrations of 2–4 g/L are rarely seen in aquatic environments, which do not seem to impede the quantitative extraction of mercury from river water matrix.

#### 3.5.4. Breakthrough Volume

It is determined by using the recommended column procedure as described above, using increasing volumes of Hg(II) solution while keeping the total amount of Hg(II) in the solution constant at 2 *µ*g. Results have shown that the maximum sample volume can be up to 2000 mL with the attainment of quantitative recovery and relative standard deviation <12%. The quantitative recovery of Hg(II) ions at high sample volumes is attributed to its high affinity and stability constant with 5Ph8HQ, in which most resins fail in Hg(II) extraction from large volumes [13]. Though a volume of 2000 mL was shown to extract effectively all target ions; a volume of 1000 mL was adopted for Hg(II) preconcentration in order to avoid long periods of extraction time (*t* > 8 hours). Considering 1000 mL as the sample volume and 4 mL as the eluent volume, an enrichment factor of 250 can be achieved.

#### 3.5.5. Analytical Performance and Applications

The optimized method was applied for the extraction of trace levels of Hg(II) ions in tap and river water samples. A volume of 1000 mL was spiked with varying quantities of 2 *μ*g and 0.5 *μ*g of Hg(II). Results shown in Table 10 show that Hg(II) spikes were quantitatively extracted from both tap and river water. As shown in pH section of the adsorption dynamics, V(II), Cu(II), Ni(II), Co(II), and Zn(II) could be simultaneously extracted with Hg(II) ions at pH 4. Previous studies have reported a simultaneous extraction of metals at pH 6–8 [35–38, 67]. For this purpose, 1000 mL of tap water was spiked with 2 *μ*g/L of a multielement mixture containing V(II), Cu(II), Ni(II), Co(II), and Hg(II) and percolated at pH 4 through 300 mg 5Ph8HQ, eluted using 4 mL of 5 M HCl + 5% CS (NH_2_)_2_, and analyzed using ICP-MS for metals and CV-AFS for Hg(II) ions. Tables 10 and 11 demonstrate simultaneous quantitative extraction of V(II), Cu(II), Ni(II),Co(II), and Hg(II) at pH 4.

The detection limit was calculated according to IUPAC [68], which is three times the standard deviation (3*б*) of eight runs of blank solution and was 0.21 ng L^−1^for Hg(II) using CV-AFS. The detection limit of ICP-MS was also calculated for V(II), Co(II), Ni(II), and Cu(II) and found to be 0.28 ng L^−1^, 0.4 ng L^−1^, 1.5 ng L^−1^, and 1.1 ng L^−1^, respectively. The relative standard deviation (RSD) of 6 runs of 2 ng mL^−1^ Hg(II) was lower than 6% indicating a good precision of the analytical method.

## 4. Conclusion

The optimized method was compared to previously developed analytical techniques for the measurements of trace levels of Hg(II). As can be seen from Table 12, the preconcentration factor is high in comparison to other methods. Moreover, LOD achieved by CV-AFS is also low as compared to other reported techniques. Even though the adsorption capacity of the studied resin is considered low, it does not seem to restrict the objectives of the analytical technique developed, that is, recovery of trace levels of mercury. High adsorption capacities of resins are rather preferable for removal studies of mercury.

In summary, 5Ph8HQ has been widely used for the preconcentration of trace levels of metals. It has been proven to be robust and exhibiting excellent affinity towards transition elements. However, it has not been examined for the extraction of mercury. Therefore, in this study, we have reported the adsorption dynamics of mercury. Results have shown that it is totally absorbed by 5Ph8HQ within the first 30 minutes of contact time with *t*_1/2_ 5 minutes, following Langmuir adsorption model. At pH 4, the affinity of mercury is unchallenged by other metals, except for Cu(II), which have shown higher *K*_*d*_ value. With these latter characteristics, 5Ph8HQ was examined for the preconcentration of trace levels of Hg(II). The developed method showed quantitative recoveries of Hg(II) with LOD = 0.21 pg mL^−1^ and RSD = 3–6% using CV-AFS.

## Figures and Tables

**Table 1:** Operating conditions ICP-MS.

Forward power (W)	1050
Nebuliser gas flow (L/min)	0,7
Auxiliary gas flow (L/min)	0,95
Cool gas flow (L/min)	13,5
Quartz plasma torch I.D. (mm)	1,5
Cone type	Pt
Glass impact bead Spray chamber temperature (°C)	3
Nebulizer type	Glass concentric
Sample uptake rate (mL/min)	1
Uptake delay (s)	35
Washout delay (s)	75
Analytes	^ 51^V, ^52^Cr, ^55^Mn, ^59^Co, ^60^Ni, ^65^Cu, ^66^Zn, ^111^Cd, ^208^Pb, ^56^Fe, ^75^As

**Table 2:** Adsorption kinetics of metals in single-metal solution.

Metal	First-order rate constants				Second-order rate constant				Intraparticular diffusion	
	*Qe* (exp) (mmol g^−1^)	*Qe* (theor) (mmol g^−1^)	*K*_1_ (min^−1^)	*R* ^2^	*Qe* (mmol g^−1^)	*K*_2_ (g mmol^−1^ min^−1^)	*R* ^2^	*t* ^1/2^	*K*_*i*_	*R* ^2^
Co	0,003394	0,0020068	0,00576	0,9988	0,00326	6,140	0,9997	49,9	0,00005	0,7041
V	0,003926	0,0000176	0,00461	0,7997	0,00392	913,816	1	0,3	0,0000006	0,5295
Pb	0,000965	0,0003075	0,00530	0,9559	0,00091	43,728	0,9999	25,2	0,000009	0,6236
Cd	0,001779	0,0000601	0,01313	0,9368	0,00178	627,455	1	0,90	0,000001	0,5016
Cu	0,003152	0,0000044	0,00668	0,9478	0,00311	5116,770	1	0,06	0,0000001	0,5123
Zn	0,003059	0,0000122	0,00299	0,9195	0,00303	819,968	1	0,40	0,0000004	0,8384
Mn	0,003640	0,0010354	0,01958	0,9961	0,00365	83,622	1	3,27	0,00005	0,3805
Ni	0,003408	0,0000186	0,00484	0,9743	0,00340	739,984	1	0,40	0,0000005	0,7674
Hg	0,000997	0,0000789	0,00507	0,9641	0,00100	186,063	1	5,39	0,000002	0,7665

**Table 3:** Adsorption kinetics of metals in multielement mixture at pH = 4.

Metal	First-order rate constants				Second-order rate constant			Intraparticular diffusion	
	*Qe* (theor) (mmol g^−1^)	*K*_1_ (min^−1^)	*R* ^2^	*Qe* (mmol/g^−1^)	*K*_2_ (g mmol^−1^ min^−1^)	*R* ^2^	*t* ^1/2^	*K*_*i*_	*R* ^2^
Co	0,00146	0,00230	0,9533	0,00255	3,283	0,991	119,61	0,00004	0,9427
V	0,00007	0,00299	0,8843	0,00390	137,896	1	1,86	0,000003	0,4423
Cu	0,00004	0,00484	0,8318	0,00315	420,290	1	0,76	0,000001	0,6093
Zn	0,00033	0,00553	0,9541	0,00096	41,447	0,9998	25,04	0,000009	0,6659
Ni	0,00161	0,00345	0,9552	0,00267	4,169	0,9987	89,83	0,00004	0,7309
Fe	0,00193	0,00161	0,7571	0,00350	2,302	0,9993	124,09	0,000008	0,3698
Hg	0,00024	0,00668	0,7452	0,00100	68,596	0,9998	14,52	0,000009	0,4446

**Table 4:** Metal distribution coefficient (*K*_*d*_) and separation factor (*α*) at pH 4 in multielement mixture.

Element	V	Co	Ni	Cu	Zn	Cd	Pb	Fe	Hg
*K*_*d*_	25.12	0.48	0.58	153.65	0.05	0.02	0.03	3.26	49.76
*α*: *K*_*d*_Hg/*K*_*d*_M^+^	1.98	102.87	85.99	0.32	931.16	2638.32	1468.79	15.28	—

**Table 5:** Selectivity of mercury at pH 4 in binary mixtures.

	V	Cu	Ni	Co	Fe	Hg
*K*_*d*_	27.011	159.800	12.829	35.836	5.260	52.376
*α*: *K*_*d*_Hg/*K*_*d*_M^+^	1.939	0.3278	4.0825	1.462	9.957	—

**Table 6:** Metal ion charge to radius ratio [8].

Ion	Oxidation state	Coordination number	Z/R
Pb	2	6	1,68
Zn	2	6	2,7
Cd	2	6	2,11
Cu	2	6	2,74
Ni	2	6	2,9
Cr	3	6	4,88
Co	2	6	2,7
V	2	6	2,53
Fe	3	6	4,651
Hg	2	6	1,96
As	3	6	5,117
Mn	2	6	2,98

**Table 7:** Distribution coefficients of metals in single-metal solution at element-optimized pH.

Element	V	Co	Ni	Cu	Zn	Cd	Pb	Mn	Hg
*K*_*d*_	137,7	799,8	59,5	160,6	18,8	199,8	2,4	999,8	52,3

**Table 8:** Langmuir, Freundlich, and D-R isotherm constants of Hg.

Metal	Langmuir isotherms parameters			Freundlich isotherms parameters			D-R isotherms parameters		
	Q_m_ (mmol g^−1^)	b (L mmol^−1^)	*R* ^2^	K_F_ (mmol g^−1^)	n	*R* ^2^	Q_m_ (mol g^−1^)	K (mol^2^ KJ^−2^)	*R* ^2^
Hg	0,0224	492,815	0,9604	0,0311	6,1463	0,9064	0.000039	1 × 10^−15^	0,9446

**Table 9:** Effects of different electrolytes on the retention of Hg(II) ions.

Electrolytes (cation/anion)	Foreign ion	Recovery (%)
Na_2_CO_3_^a^	8,75	95,4 ± 0,15
Na_3_C_6_H_5_O_7_^a^	8,64	95,9 ± 0,16
KNaC_4_H_4_O_6_^a^	20,76	96,7 ± 0,21
Na_2_C_2_O_4_^a^	30,07	94,2 ± 0,11
KCN^a^	40,28	86,7 ± 0,22
Humic acid^b^	0,42	96,3 ± 0,14
SO_4_^2−^ ^a^	1,04	97,0 ± 0,37
Cl^−^^a^	2,82	95,9 ± 0,26
NO_3_^−^^a^	1,61	96,9 ± 0,22
F^−^^a^	0,53	94,5 ± 0,14
Co^2+^ ^a^	1,36	99,1 ± 0,11
Fe^3+^ ^a^	1,43	97,0 ± 0,91
Ni^2+^ ^a^	1,36	99,1 ± 0,23
V^4+^ ^a^	1,57	99,2 ± 0,15
Cu^2+^ ^a^	1,26	98,3 ± 0,85

a: mmol L^−1^; b: g L^−1^.

**Table 10:** Analytical results for the determination of Hg(II) in natural water samples by CV-AFS.

Water samples	Concentration *µ*g L^−1^		Recovery (%)
	Added	Found^a^	
Tap water	0	0,774 ± 0,034	
	0,5	1,274 ± 0,082	100
	2	2,769 ± 0,066	99,8

River water	0	0,382 ± 0,069	
	0,5	0,882 ± 0,085	100
	2	2,380 ± 0.055	99,9

^a^The value following “±” is the standard deviation (*n* = 2).

**Table 11:** Simultaneous determination of metals in natural water samples by ICP-MS at pH 4.

Water samples	Element	Concentration *µ*g L^−1^		Recovery (%)
		Added	Found^a^	
Tap water	V	0	0,320 ± 0,106	
		2	2,315 ± 0,21	99,8
	Co	0	0,059 ± 0,004	
		2	2,041 ± 0,02	99,1
	Ni	0	4,392 ± 0,037	
		2	6,172 ± 0,05	96,5
	Cu	0	0,762 ± 0,137	
		2	2,759 ± 0,32	99,9

River water	V	0	0,606 ± 0,428	
		2	2,602 ± 0,52	99,8
	Co	0	0,041 ± 0,014	
		2	2,030 ± 0,05	99,4
	Ni	0	2,964 ± 0,377	
		2	4,790 ± 0,52	96,4
	Cu	0	0,066 ± 0,42	
		2	2,065 ± 0,32	99,9

^a^The value following “±” is the standard deviation (*n* = 2).

**Table 12:** Comparison of figures of merits for different preconcentration methods for Hg(II) ion.

Chelating resin	DT^a^	pH	Capacity (mmol·g^−1^)	LOD^b^ (pg · mL^−1^)	PF^c^	Reference^d^
(1) poly(acrylamide) grafted onto cross-linked poly(4-vinyl pyridine) (P4-VP-*g*-PAm)	AFS	1–8	4.1	2	20	[9]
(2) Silica gel/2-thiophenecarboxaldehyde	AAS	2	2	4.75	1000	[10]
(3) Sodium dodecyl sulphate-coated magnetite nanoparticles/Michler's Thioketone complexes	ICP-AES	3	—	40	1230	[11]
(4) Magnetic nanoparticles doped with 1,5-diphenylcarbazide	CV-AAS	6	0.22	160	100	[12]
(5) Poly-allylthiourea	CV-AAS	2	1.1	80	160	[13]
(6) Silica gel/2-(2-oxoethyl)hydrazine	ICP-AES	4	0.18	100	50	[14]
(7) Polyaniline (PANI)	gold trap-CVAAS	1–12	0.49	0.05	120	[15]
(8) Hg(II)-Ionic imprinted polymers-thymine	AFS	8	0.014	30	200	[16]
(9) Silica gel/cysteine	CV-AAS	1–7	0.69	1.5	40	[17]
(10) Alumina/dimethylsulfoxide	AAS	1-2	381	—	1000	[18]
(11)C18/Isopropyl 2-[(isopropoxycarbothioly)disulfany]ethane thioate	CV-AAS	3–7	0.015	5	>150	[19]
(12) Hg(II)-imprinted ion polymer	CV-AAS	8	0.205	50	200	[20]
(13) Agar powder modified with 2-mercaptobenzimidazole	CV-AAS	2-3	1.89	20	100	[21]
(14) Hg(II)-imprinted thiol-functionalized mesoporous sorbent	ICP-AES	4–8	0.022	390	150	[22]
(15) Silica gel immobilized 5-phenylazo-8-hydroxyquinoline	CV-AFS	4	0.027	0.21	>250	This work

^a^Detection technique; ^b^limit of detection; ^c^preconcentration factor; ^ d^reference.
